# Evaluation of Osteoporosis Perception Among Saudi Arabian Premenopausal Women: A Cross-Sectional Survey Study Using the Osteoporosis Knowledge Assessment Tool (OKAT)

**DOI:** 10.7759/cureus.45191

**Published:** 2023-09-13

**Authors:** Abdulrahman Alghamdi, Omar Abdullah Almutairi, Rakan Abu Alqam, Abdulaziz Jambi, Hattan S Alharthi, Khalid binhamran, Hala Mosli

**Affiliations:** 1 Hematology, Faculty of Medicine, King Abdulaziz University, Jeddah, SAU; 2 Medicine, Faculty of Medicine, King Abdulaziz University, Jeddah, SAU; 3 Endocrinology and Metabolism, Faculty of Medicine, King Abdulaziz University, Jeddah, SAU; 4 Endocrinology, King Abdulaziz University Hospital, Jeddah, SAU

**Keywords:** health beliefs, osteoporosis knowledge assessment tool "okat", perception, premenopausal women, osteoporosis

## Abstract

Introduction: Osteoporosis is a major clinical problem that affects the whole population, especially women. It is a condition that is becoming more prevalent with aging. The increase in bone fragility associated with the disease can lead to fractures, even from minor trauma. Our goal is to evaluate the extent of knowledge about osteoporosis and perceptions of it among premenopausal women in Saudi Arabia.

Method: This descriptive cross-sectional study was conducted among premenopausal women during the months of June and July 2021. We distributed an online questionnaire on social networking sites and applications that Saudi women use on a daily basis. We used the Osteoporosis Knowledge Assessment Tool (OKAT) to evaluate the responses. Result: A total of 661 women took part in our study; 159 of them were excluded because they had either reached the menopausal period or had already been diagnosed with the disease. The overall perception of osteoporosis is noticeably poor, as only 55% of respondents had an acceptable level of knowledge. In addition, we found a statistically significant association between education level and knowledge level (p-value = 0.044). Conclusion: The findings revealed that more than half the participants scored "acceptable" in terms of their understanding of the disease, which indicates a serious awareness gap. This outcome demonstrates the necessity of increasing community awareness about osteoporosis to reduce potential harm and the financial burden of healthcare.

## Introduction

Osteoporosis is a multifactorial, progressive chronic condition that results in slow bone weakening and loss, microscopic tissue degradation, and an elevated risk of fracture [1]. It is referred to as a systemic skeletal disorder that is characterized by low bone density and the degradation of the main bulk of the bone, which leads to more porous bone and an elevated risk of fracture, even from minor slips, trips, or accidents [2,3]. While osteoporosis cannot be cured, it can be prevented by quitting smoking, drinking less alcohol, eating enough calcium and vitamin D, avoiding falls, and increasing physical activity overall at all ages [4]. Additionally, osteoporosis is regarded as the second-most important health concern in developed nations after heart disease [5]. More than 8.9 million fractures occur annually, affecting an estimated 200 million women worldwide [6]. According to the International Osteoporosis Foundation, the risk of fracture rises with age, with 20% of men and 50% of women having experienced an osteoporosis-related fracture in their lifetime [7]. Postmenopausal women and the elderly are typically the groups most affected by the condition.

A previous study revealed that the high prevalence of osteoporosis in Saudi Arabia is causing serious concerns about bone health. An analysis revealed that 34% of healthy Saudi women and 30.7% of healthy Saudi men between 50 and 79 are osteoporotic, with the prevalence expected to rise as the country’s life expectancy increases [8]. A cross-sectional study was conducted in 2018 at King Abdullah City for Female Students, Al-Imam Mohammad ibn Saud Islamic University, to assess young women’s general perceptions and health beliefs regarding osteoporosis; the results demonstrate that 79.4% of the 1,012 respondents who were surveyed lacked sufficient perceptions of the illness [9]. According to a 2017 study conducted in Enugu, osteoporosis awareness in Nigeria was low and unrelated to gender, marital status, or level of education [10]. Furthermore, the results from an Indian study published in 2019 found that 60% of the participants (a total of 182 postmenopausal women) had a low level of perception regarding osteoporosis, an unsatisfactory outcome [11]. A Pakistani study published in 2008 found that younger women knew relatively little about osteoporosis compared to older women [12]. This is, as far as we know, the first study conducted among premenopausal women. In light of this existing literature, our study aims to evaluate premenopausal women’s awareness regarding osteoporosis risk factors, symptoms, risk of fractures, and management in Saudi Arabia to improve the relevant preventive services since these patients bring a significant burden to the healthcare system.

## Materials and methods

This cross-sectional study was conducted in 2021 at King Abdulaziz University Hospital (KAUH), Jeddah, Saudi Arabia. This study focuses on Saudi Arabian women between the ages of 16 and 51 who are residents or citizens of the country. Women who have gone through menopause or who have previously been diagnosed with osteoporosis were excluded. During June and July 2021, a Google Forms (Google LLC, Mountain View, CA, USA) survey was distributed on social networking applications and websites that Saudi Arabian women often use. All respondents gave their consent before answering the questionnaire, and they then had the option of choosing the language in which they preferred to respond. Following that, the survey asked questions regarding demographics. These questions addressed information such as nationality, marital status, smoking status, age at menarche, regularity of menstruation, history of steroid or hormonal consumption, work environments, whether or not they were related to the healthcare system, place of employment, and educational level. We asked two additional questions: "Do you have osteoporosis?" and "Have you reached the menopausal period?" to ensure that every woman who answered the survey was a member of the target population. Any women who responded positively to these questions were omitted.

The responses were then evaluated using the Osteoporosis Knowledge Assessment Tool (OKAT), a questionnaire that measures the extent of knowledge on osteoporosis created and adopted in an Australian study published in 2003 [1]. The questionnaire was translated into Arabic in a Syrian article published in 2013 [3]. Specialists tested and verified the translation process, and several medical translators worked to ensure the accuracy of understanding and comprehension for all participants. The questions and answers were collected in a Microsoft Excel (Microsoft Corp., Redmond, WA, USA) spreadsheet. We then encoded the questions and answers in the statistical analysis tool SPSS Statistics version 26 (IBM Corp., Armonk, NY, USA) to make them easier to understand. In the OKAT, we coded the correct answer with "1" in each item and the wrong answers and "I don’t know" answers with "0". Then, we added the participants’ correct answers and calculated the total knowledge of each person as a new variable in the statistical program, with the ideal result being 20 for the person whose answers were all correct. To divide and categorize knowledge ratios, we adopted four categories based on the correct answers to the questions that aim to measure the extent of knowledge about the disease: any person who answered five questions or fewer was classified as having poor knowledge; more than five but fewer than 10 correct answers were classified as having acceptable knowledge; 10 to 15 correct answers were classified as average; and anyone who answered 15 or more questions correctly was rated as having good knowledge.

## Results

We carried out this study to evaluate the attitudes and awareness of the premenopausal women's population in Saudi Arabia, as there is little available data on their health beliefs or views of osteoporosis. We received 661 responses from women who reside in Jeddah, Saudi Arabia; 159 of them were excluded because they had either reached menopause or had already been diagnosed with the disease. Following that, 502 women were enrolled in the study. Table 1 shows their varied demographic characteristics. The bulk of the participants were between the ages of 16 and 29 (54.8%). In addition, over 90.4% of the participants said that they did not smoke. Around 55% of the respondents were single; those holding a bachelor’s degree made up over 60% of the population; and 93.4% of them were Saudi.

**Table 1:** Sociodemographic characteristics of participants

Sociodemographic data		Number	Percentage
Age group	16 to 29	275	54.8%
	30 to 39	70	13.9%
	40 to 49	138	27.5%
	50 to 51	19	3.8%
Nationality	Saudi	469	93.4%
	Non-Saudi	33	6.6%
Education level	Bachelors	303	60.4%
	High school	135	26.9%
	Masters	21	4.2%
	PhD	11	2.2%
	Diploma	32	6.4%
Marital status	Single	276	55%
	Married	202	40.2%
	Divorced or widow	24	4.8%
Do you have osteoporosis?	Yes	0	0%
	No	502	100%
Have you reached the menopausal period?	Yes	0	0%
	No	502	100%
Do you have regular menses?	Yes	394	78.5%
	No	108	21.5%
Your age at puberty	Less than 13	184	36.7%
	13-15	295	58.8%
	More than 16	23	4.6%
Your smoking status	Yes	48	9.6%
	No	454	90.4%
Did you take any type of steroid or hormone?	yes	69	13.7%
	no	433	86.3%
What is your place of work?	Hospital	85	16.9%
	Faculty or schools	183	36.5%
	I do not work	165	32.9%
	Others	69	13.7%
The work environment in which you work or study	Non-health professional	306	61%
	Health professional	196	39%

None of the items in the questions had any reported missing values. Over 93% of the population knew that osteoporosis can lead to bone fractures; however, just 5% were aware that it can induce symptoms such as soreness before fractures occur. Nearly 53% were aware that having an osteoporotic family member increases one’s risk of developing the condition themselves in the future. In addition, only around 9.2% of the participants were aware of the impact of bone loss in the 10 years following the onset of menopause. When they were asked about the role of hormone therapy, 26.9% knew that it aids in preventing bone loss following menopause. While speaking about dietary intake and supplements, more than half of the population chose "two glasses of milk" as being enough and "sardines" and "broccoli" as good resources for those who cannot tolerate dairy products (59% and 67%, respectively). When questioned about the importance of exercise, only 15.1% of the participants knew that not all forms of exercise are good for preventing osteoporosis (Table 2).

**Table 2:** Osteoporosis Knowledge Assessment Tool (OKAT)

OKAT Questions	Correct answer	Number of people who answered correctly	Percentage
1: Osteoporosis leads to an increased risk of bone fractures	True	467	93%
2: Osteoporosis usually causes symptoms (e.g., pain) before fractures occur	False	25	5%
3: Having a higher peak bone mass at the end of childhood gives no protection against the development of osteoporosis in later life	False	99	19.7%
4: Osteoporosis is more common in men	False	345	68.7%
5: Cigarette smoking can contribute to osteoporosis	True	273	54.4%
6: White women are at highest risk of fracture compared to other races	True	134	26.7%
7: A fall is just as important as low bone strength in causing fractures	True	367	73.1%
8: By age 80, the majority of women have osteoporosis	True	357	71.1%
9: From age 50, most women can expect at least one fracture before they die	True	164	32.7%
10: Any type of physical activity is beneficial for osteoporosis	False	76	15.1%
11: It is easy to tell whether I am at risk of osteoporosis by my clinical risk factors	True	225	44.8%
12: Family history of osteoporosis strongly predisposes a person to osteoporosis	True	268	53.4%
13: An adequate calcium intake can be achieved from two glasses of milk a day	True	296	59%
14: Sardines and broccoli are good sources of calcium for people who cannot take dairy products	True	337	67.1%
15: Calcium supplements alone can prevent bone loss	False	289	57.6%
16: Alcohol in moderation has little effect on osteoporosis	True	95	18.9%
17: A high salt intake is a risk factor for osteoporosis	True	174	34.7%
18: There is a small amount of bone loss in the 10 years following the onset of menopause	False	46	9.2%
19: Hormone therapy prevents further bone loss at any age after menopause	True	135	26.9%
20: There are no effective treatments for osteoporosis available in Saudi Arabia	False	177	35.3%

The majority of responses to the OKAT questionnaire ranged between poor knowledge and acceptable knowledge (15.3% and 55%, respectively); 28.3% of the individuals had average knowledge, leaving just 1.4% with good knowledge (Table 3). We examined the socio-demographic elements that affect people’s awareness of the osteoporosis condition, and we found that there is a highly significant correlation between work environment and osteoporosis knowledge (p-value = 0.0001). Additionally, we discovered a strong correlation between the level of education and the understanding of osteoporosis (p-value = 0.044). Moreover, the survey showed a significant relationship between the workplace and one’s knowledge of osteoporosis (p-value = 0.0001) (Table 4).

**Table 3:** Categories of answers related to OKAT OKAT: Osteoporosis Knowledge Assessment Tool

Categories	Number of persons	Percentage
Poor	77	15.3%
Acceptable	276	55%
Average	142	28.3%
Good	7	1.4%
Total	502	100%

**Table 4:** Differences between the different factors assessed by OKAT OKAT: Osteoporosis Knowledge Assessment Tool

Factor	p=value
Age group	0.0001
Workplace environment	0.044
Level of education	0.0001
Place of work	0.211
Regularity of menses	0.942

## Discussion

In our study, we investigated how well premenopausal women were aware of and knew about osteoporosis. Of the 502 participants, the majority had an acceptable level of perception (55%), but, surprisingly, only 1.4% had good knowledge, which is an extremely low percentage. Our findings coincide with a study carried out in Riyadh, Saudi Arabia, which found that 79.4% of the 1,012 participants had inadequate awareness about osteoporosis [9]. Additionally, both a 2012 study in Aseer, Saudi Arabia, and a study at the security forces hospital in Riyadh found that perceptions were insufficient, particularly among young women [13,14]. In addition, numerous other studies in Turkey, Vietnam, and India demonstrate that premenopausal women lack basic information on osteoporosis [15,16]. According to a study conducted in New Zealand, age has an impact on the level of knowledge, as older women (40 to 49 years old) scored the highest mean score (17.3) of osteoporosis knowledge (SD 4.0) [17]. Another study carried out in Riyadh found a statistically significant relationship between age and level of knowledge [14], indicating that older women had higher scores than younger women and had better knowledge about osteoporosis. Additionally, a 2006 study from Pakistan found that younger women had lower levels of knowledge than older women, which contrasts with our finding that age has no impact on a person’s level of perception and beliefs [18].

Furthermore, our findings revealed that the level of education influences the level of knowledge of osteoporosis (p-value = 0.044). These results are supported by other studies conducted in Saudi Arabia and Turkey [13,19,20]. However, one study opposes our finding and argues that there are no statistically significant differences between the levels of knowledge and education [9]. In alignment with a recent study conducted in Saudi Arabia, we discovered that a family history of osteoporosis was not related to the level of knowledge regarding osteoporosis. It is important to understand that modifying some health behaviors, like eating habits and physical activity, can help prevent osteoporosis. Only 76 (15.1%) of our 502 participants agreed that any form of physical activity is beneficial for osteoporosis, which is similar to a recent study conducted in Riyadh, which also found a low percentage of the respondents providing the correct answer (only 20.7%) [9].

Our study has a few limitations, such as the cross-sectional research design and the convenience sampling technique for choosing the participants, that may have led to some bias. Future research must involve a greater number of premenopausal women from numerous areas of Saudi Arabia with a range of educational backgrounds.

## Conclusions

Our study demonstrated that young Saudi women lack the knowledge necessary to prevent osteoporosis. These findings suggest the need for more preventive programs that raise the general public’s awareness and understanding of this disease, which will reduce the financial burden on global health organizations. We recommend that future researchers seek out ways to increase their sample size as much as possible and cover more areas of Saudi Arabia.
